# Epidermoid cyst of the penis: A case report

**DOI:** 10.1016/j.ijscr.2025.111181

**Published:** 2025-03-18

**Authors:** Salim Lachkar, Imad Boualaoui, Ahmed Ibrahimi, Syrine Hamada, Hachem El Sayegh, Yassine Nouini

**Affiliations:** a Department of Urology A, Ibn Sina University Hospital, Rabat, Morocco; b Department of Dermatology, Ibn Sina University Hospital, Rabat, Morocco

**Keywords:** Epidermoid cyst, Penile lesions, Surgical excision, Follicular keratosis, Benign tumors

## Abstract

**Introduction:**

Epidermoid cysts are benign, keratin-filled lesions that rarely occur in the genital region. Diagnosis is primarily clinical, but histopathological examination is essential for confirmation. This case highlights a rare occurrence of an epidermoid cyst on the penis, emphasizing clinical features, diagnostic approach, and treatment outcomes.

**Case presentation:**

A 35-year-old male presented with a non-tender, firm, well-circumscribed lesion measuring 2.5 cm × 1.8 cm located on the middle third of the left lateral aspect of the penis. Over eight months, the mass grew, causing pruritus, sexual discomfort, and self-image concerns, without pain or infection. Histopathological analysis confirmed the diagnosis of an epidermoid cyst, with stratified squamous epithelium and keratin-filled cystic spaces.

**Discussion:**

Although rare in the genital area, epidermoid cysts must be considered in the differential diagnosis of penile lesions. Clinical presentation, including a well-defined, mobile mass, is suggestive of the condition. Histopathological features, such as a cyst lined with squamous epithelium and keratin, are key to distinguishing it from other conditions like squamous cell carcinoma. Surgical excision with a margin is the treatment of choice, and recurrence is uncommon when performed adequately.

**Conclusion:**

Epidermoid cysts of the penis are benign lesions with a favorable prognosis following surgical excision. This case demonstrates the importance of accurate diagnosis and management to avoid misdiagnosis and ensure successful treatment outcomes. The patient showed no recurrence at the 6-month follow-up.

## Highlights

• Rare but benign; requires thorough differential diagnosis
• Surgical excision is curative in most cases.
• Postoperative surveillance is crucial to avoid recurrence.
• Histopathological confirmation is essential to ensure accurate diagnosis and exclude malignancies.

## 1 Introduction

Epidermoid cysts (EC) are slow-growing, benign lesions resulting from the entrapment of epidermal cells in the dermis [1]. While they are commonly found on the face, scalp, and trunk, penile localization is extremely rare, representing between 0.01 % and 0.1 % of penile masses [2]. This prevalence is an estimate, as the incidence is difficult to assess due to the rarity of cases, with fewer than 55 published cases of penile EC worldwide [3]. This report examines the clinical presentation, diagnosis, treatment, and surgical techniques of penile EC, comparing it with the latest 10 case reports [[2], [3], [4], [5], [6], [7], [8], [9], [10], [11]] on surgical methods and follow-up outcomes.

## 2 Case presentation

A 35-year-old married male, with no significant medical history, no history of multiple sexual partners, and a history of ritual circumcision in childhood, presented with an asymptomatic mass on the lateral left side of the penis, which had been present since 2019. Over the past 8 months, the mass gradually increased in size and was associated with intense pruritus. The patient reported discomfort during sexual intercourse and a negative impact on his self-image. There was no pain, discharge, or signs of infection.

**Fig. 1:**
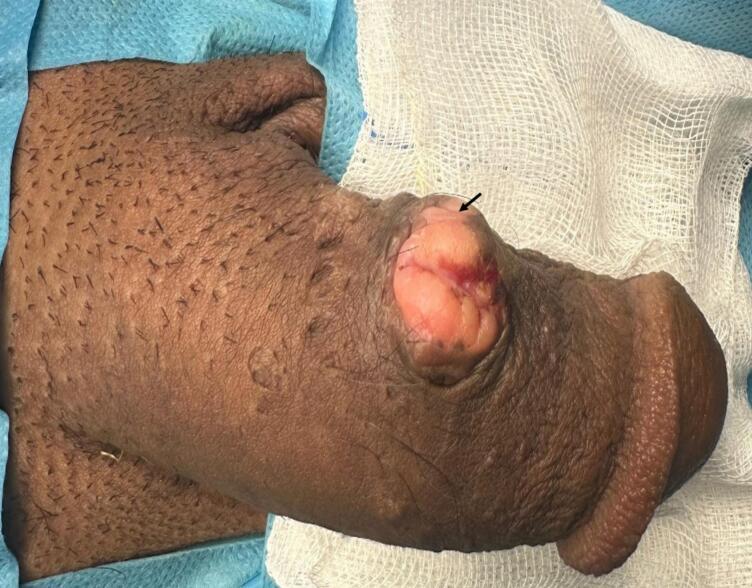
Preoperative appearance of the lesion: The lesion appears as a well-circumscribed, firm mass located on the middle third of the left lateral aspect of the penis, with no signs of ulceration (black arrow).

On clinical examination, a firm, mobile, non-tender, well-circumscribed, and superficial subcutaneous lesion measuring 2.5 cm × 1.8 cm was observed on the middle third of the left lateral aspect of the penis. No signs of inflammation, such as erythema or ulceration, were noted, and no palpable inguinal lymphadenopathy was present. The corpora cavernosa were palpated freely, and the spongy body was palpated distally (Fig. 1).

Serological tests for HIV, HBV, HCV, VDRL, and TPHA were negative, excluding common infectious causes. At this stage, the differential diagnosis included epidermoid carcinoma, Bowen's disease, adnexal tumor, leiomyoma, and cutaneous tuberculosis. A biopsy was necessary to confirm the diagnosis.

A cutaneous biopsy was performed, consisting of two skin flaps measuring 1.5 × 0.3 × 0.3 cm and 1.6 × 0.9 × 0.9 cm. Histopathological examination revealed acanthotic epidermal lining with orthokeratotic hyperkeratosis and focal parakeratosis. Spongiosis was noted, and the underlying dermis contained a cystic formation lined by squamous epithelium without cytonuclear atypia, but with keratin flakes. The cyst was surrounded by a moderate polymorphic inflammatory infiltrate, both perivascular and interstitial. No pathogenic agents were identified with PAS staining. The final diagnosis was an epidermoid cyst, with spongiotic dermatitis likely being an artifact due to secondary irritation from the patient's frequent scratching. The spongiosis was localized and not accompanied by signs of infection or primary inflammatory pathology. This is not typically associated with epidermoid cysts and suggests a reaction to mechanical irritation. Therefore, further radiological assessment was deemed unnecessary.

**Fig. 2:**
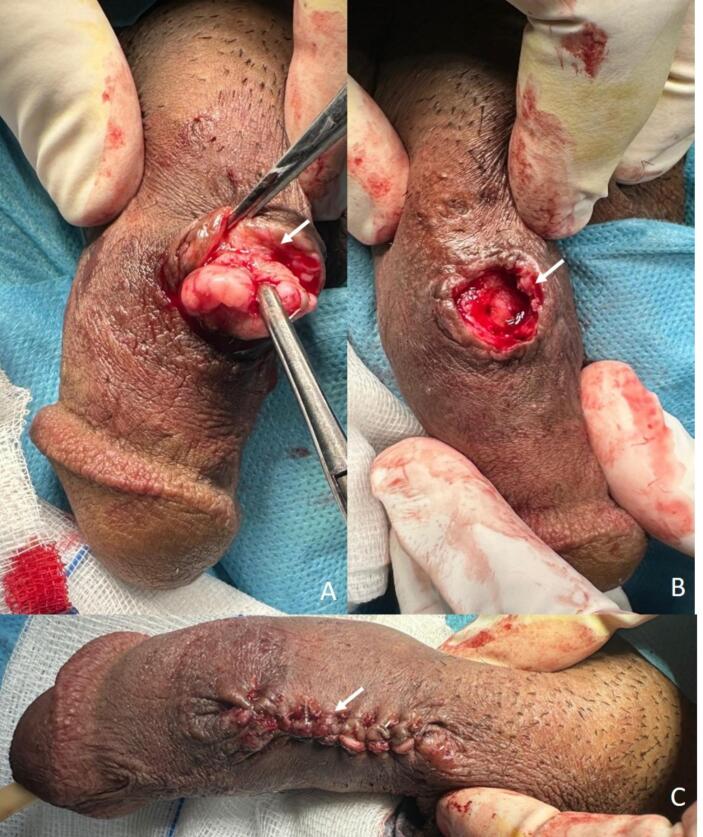
Intraoperative findings: (A) Lesion excision in progress, revealing well-defined margins with meticulous dissection (white arrow); (B) post-excision view of the penis, demonstrating an intact surgical site with preserved corpora cavernosa (white arrow); (C) final appearance after wound closure (white arrow).

The lesion was surgically excised under local anesthesia with sedation. The procedure was performed through an elliptical incision following the natural skin lines, ensuring complete excision of the lesion along with the surrounding subcutaneous tissue. Careful hemostasis was achieved using electrocautery, and the wound was closed in layers with absorbable sutures for the deep tissue and interrupted absorbable sutures for the skin. The excised specimen measured 2.7 cm by 2.0 cm (Fig. 2).

**Fig. 3:**
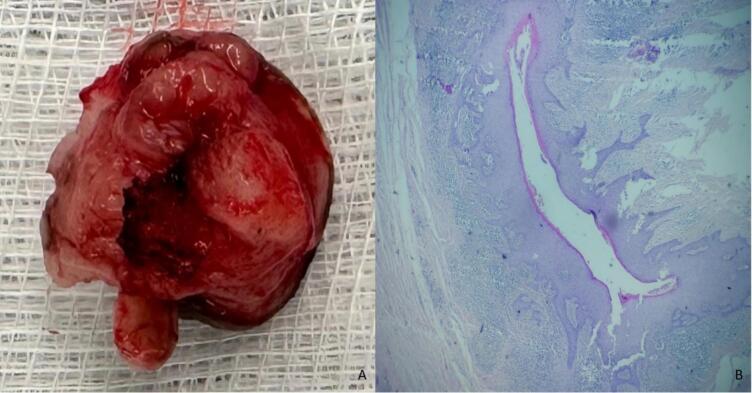
Macroscopic and histopathological findings: (A) Macroscopic view of the excised specimen, showing a well-circumscribed, encapsulated mass with a smooth outer surface; (B) histopathological examination at low magnification (HE staining) showing cystic structure lined by stratified squamous epithelium with lamellated keratin in the lumen and no atypia.

Definitive histopathological analysis showed a dermal cystic formation with a cutaneous lining. The lining was bordered by regular, mature squamous epithelium, and a well-defined granular layer was noted. The cyst lumen contained concentric lamellae of keratin. The dermis was loosely fibrous, with rare mononuclear inflammatory cells composed primarily of mature lymphocytes, traversed by a fine capillary network. Rare atrophic follicles were noted, surrounded by concentric fibrosis. The final diagnosis confirmed an epidermoid cyst, associated with follicular keratosis (Fig. 3).

**Fig. 4:**
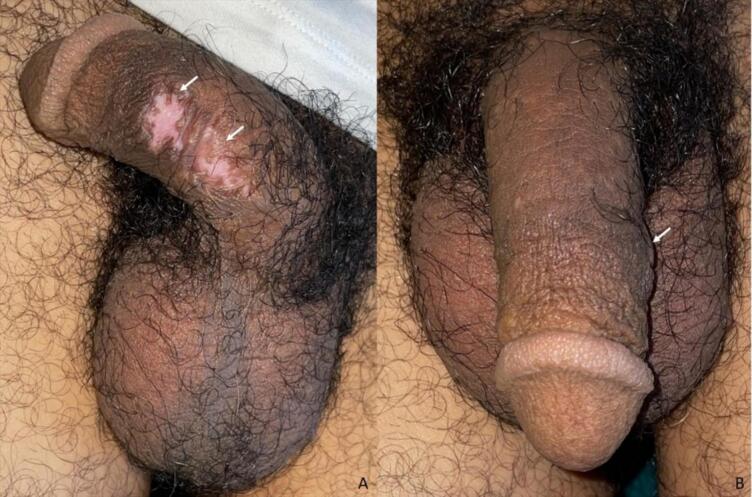
Appearance at 6 months. Lateral and frontal views showing the healed surgical site with no signs of recurrence, smooth skin, and normal penile appearance (A: lateral, B: frontal).

The postoperative period was uneventful, with complete healing observed in three weeks. At the six-month follow-up, no recurrence was noted, and the patient reported a high level of satisfaction with both functional and aesthetic outcomes (Fig. 4).

## 3 Discussion

EC are benign lesions derived from the epidermis, accounting for approximately 1 % of cutaneous tumors and up to 85–90 % of benign cystic skin tumors [4]. Penile localization of EC is extremely rare, representing between 0.01 % and 0.1 % of penile masses [2]. This prevalence is an estimate, as the incidence is difficult to assess due to the rarity of cases, with fewer than 55 published cases of penile EC [3].

EC primarily affect men, with a male-to-female ratio of 3:1. The peak incidence is between 30 and 50 years, although cases span from infancy to old age. Prevalence ranges from 0.1 % to 0.7 % [5]. Most cysts are found on the trunk (35 %), head and neck (25 %), and extremities (20 %). Genital involvement is rare, accounting for <0.01 % of cases, based on reviews of over 2500 skin lesions [12].

EC occur when epidermal cells are trapped within the dermis, resulting in a gradual buildup of keratin in a cystic cavity, which is lined by stratified squamous epithelium with a defined granular layer [1]. The pathogenesis of these cysts can be attributed to three main mechanisms. Post-traumatic epidermal inclusion is responsible for approximately 50 % of cases, often following penetrating injuries, surgical procedures, or chronic friction that displaces epidermal cells into the dermis [2]. Obstruction of cutaneous appendages, such as sebaceous glands and hair follicles, accounts for nearly 30 % of cases [13]. This typically occurs in conditions like acne vulgaris, hidradenitis suppurativa, or pilonidal disease, where follicular occlusion leads to the accumulation of cellular debris and cyst formation [14]. Congenital or genetic factors contribute to around 20 % of cases, with EC sometimes arising in association with developmental anomalies such as branchial cleft remnants or underlying genetic conditions like Gardner's syndrome, which involves multiple epidermoid cysts, desmoid tumors, and colorectal polyposis [15]. At the molecular level, studies have revealed mutations in the PTCH1 gene, a critical regulator of the Hedgehog signaling pathway, in certain congenital forms of epidermoid cysts [5]. These PTCH1 mutations result in abnormal cell proliferation and differentiation, making individuals more susceptible to the formation of multiple cysts. Additionally, disruptions in other key signaling pathways, such as Wnt/β-catenin and TGF-β, have been implicated in the altered proliferation and differentiation of keratinocytes, particularly in sporadic cases [15].

Follicular keratosis, also known as keratosis pilaris, is a condition characterized by abnormal keratinization of hair follicles [16]. It is typically observed on the upper arms, thighs, face, and buttocks, but genital involvement is rare [3]. When it does occur, it is usually asymptomatic and manifests as small, rough, keratotic papules. The exact prevalence of follicular keratosis in the genital region is not well established, but it is considered exceedingly uncommon, often in association with other dermatological conditions [16]. Although benign, follicular keratosis must be differentiated from more serious conditions, including malignancies, during evaluation [3].

Imaging is typically unnecessary for well-circumscribed superficial lesions, as clinical examination and histopathology are usually sufficient [14]. However, if malignancy is suspected or if the lesion has atypical characteristics, further investigation may be needed. In such cases, high-frequency ultrasound (>15 MHz) is commonly used, revealing an anechoic or hypoechoic homogeneous structure with well-defined walls and no vascular flow on Doppler, suggesting a benign nature [1]. This imaging modality is helpful for deeper or more complex cysts. MRI may be considered if malignancy is suspected, as it shows characteristic T2 hyperintensity, which could indicate tumor tissue or inflammation [6].

The differential diagnosis for EC includes various conditions.

Frequent benign lesions are condyloma acuminata (HPV), accounting for up to 70 % of benign penile lesions, most commonly on the glans (50 %), shaft (30 %), and coronal sulcus (20 %) [17]. Primary syphilis, affecting <5 % of penile lesions, typically presents as a chancre on the glans (50 %), shaft (30 %), or prepuce (20 %). Sebaceous cysts make up 5–10 % of benign masses, typically on the shaft (60–70 %) or glans (10–15 %) [6]. Lipomas, also 5–10 % of benign masses, are mainly located on the shaft (70 %) or scrotum (20 %) [7]. Pilonidal disease is rare on the penis but can occur on the shaft in under 5 % of cases [12]. Leiomyomas, representing 1–2 % of benign penile tumors, are found mostly on the shaft (80 %) or glans (10 %) [18].

Rare but significant lesions include squamous cell carcinoma (95 % of penile cancers), most often on the glans (50 %), prepuce (30 %), or shaft (20 %) [19]. Bowen's disease occurs in 1–5 % of penile cancers and may progress to invasive carcinoma [11]. Penile melanoma, <1 % of penile cancers, presents as a pigmented lesion on the glans (60 %), shaft (30 %), or prepuce (10 %) [19].

Very rare conditions include cutaneous tuberculosis (<1 % of extrapulmonary tuberculosis), presenting as a painless lesion on the glans (60 %) or shaft (40 %) [7]. Rare genetic conditions like Gardner's syndrome and basal cell nevus syndrome may cause EC on the penis, typically on the shaft (60 %) or glans (20 %), requiring monitoring for associated malignancies, including colorectal cancer [15].

We opted for surgical excision, the most common approach in recent case reports [2,[4], [5], [6], [7], [8], [9], [10], [11]]. Surgical excision is the primary treatment for EC, with a success rate of 98–100 % [14]. Elliptical excision is the standard technique used in 90 % of cases, while marsupialization is used for cysts larger than 5 cm. CO2 laser excision, associated with a recurrence rate of <3 %, is employed to reduce scarring [6]. Postoperative complications are rare, including infections (2–5 %), hypertrophic scarring (3–5 %), and transient erectile dysfunction (1 %) [11].

Histopathological examination confirms the diagnosis of EC, which feature stratified squamous epithelium without atypia, distinguishing them from malignancies like squamous cell carcinoma [7]. The cyst contains lamellated keratin and, in 20–40 % of cases, a moderate inflammatory infiltrate [12]. In the current case, histopathology showed orthokeratotic hyperkeratosis, a polymorphic infiltrate, and no atypia, confirming the lesion's benign nature. Unlike dermoid cysts, EC lack adnexal structures and have a lining of 6–15 squamous cell layers. The cyst contents consist of keratin, not sebaceous material, and inflammation is common in ruptured cases, leading to granuloma formation [1].

Our favorable postoperative outcomes, align with results seen in similar cases [[2], [3], [4], [5], [6], [7], [8], [9], [10], [11]]. The prognosis is excellent, with a recurrence rate of <5 %, and no malignant transformation observed after a median follow-up of 5 years [10]. Functional and aesthetic satisfaction is reported in 95 % of cases [1]. Recurrence occurs in 2–5 % of cases, especially after incomplete excision [10].

## 4 Conclusion

Penile EC are rare lesions. Accurate diagnosis is vital to rule out malignancies. Complete excision ensures excellent functional and aesthetic outcomes. Follow-up beyond six months is crucial to monitor for rare recurrences and ensure early detection of complications.

## CRediT authorship contribution statement

Salim Lachkar: Study concept and design, data analysis, writing, and critical revision of the manuscript.
Imad Boualaoui: Data collection, literature review, and drafting of the manuscript.
Ahmed Ibrahimi: Data collection, literature review.
Syrine Hamada: Data analysis, supervision and critical revision.
Hachem El Sayegh: Supervision, critical revision, and final approval of the manuscript.
Yassine Nouini: Supervision, critical revision, and final approval of the manuscript.

## Consent

The patient provided informed consent after receiving detailed information regarding the study and its implications.

## Ethical approval

Ethical approval was obtained from our institution. Informed consent was provided by the patient for the publication of case details and images.

## Guarantor

Lachkar Salim.

## Research registration number

Not applicable to this case report.

## Methods

This case report has been reported in line with the SCARE criteria [20].

## Funding

This research and the publication expenses were solely supported by the author, and no specific grant was received from any public, commercial, or non-profit sectors.

## Declaration of competing interest

None.
